# Correction: Anti-schistosomal activity and ADMET properties of 1,2,5-oxadiazinane-containing compound synthesized by visible-light photoredox catalysis

**DOI:** 10.1039/d4md90044h

**Published:** 2024-10-24

**Authors:** Kennosuke Itoh, Hiroki Nakahara, Atsushi Takashino, Aya Hara, Akiho Katsuno, Yuriko Abe, Takaaki Mizuguchi, Fumika Karaki, Shigeto Hirayama, Kenichiro Nagai, Reiko Seki, Noriko Sato, Kazuki Okuyama, Masashi Hashimoto, Ken Tokunaga, Hitoshi Ishida, Fusako Mikami, Kofi Dadzie Kwofie, Hayato Kawada, Bangzhong Lin, Kazuto Nunomura, Toshio Kanai, Takeshi Hatta, Naotoshi Tsuji, Junichi Haruta, Hideaki Fujii

**Affiliations:** a Laboratory of Medicinal Chemistry, School of Pharmacy, Kitasato University 5-9-1 Shirokane, Minato-ku Tokyo 108-8641 Japan itok@pharm.kitasato-u.ac.jp; b Medicinal Research Laboratories, School of Pharmacy, Kitasato University 5-9-1 Shirokane, Minato-ku Tokyo 108-8641 Japan; c Department of Material Science, Graduate School of Science, Josai University 1-1 Keyakidai Sakado Saitama 350-0295 Japan; d Division of Liberal Arts, Center for Promotion of Higher Education, Kogakuin University 2665-1 Nakano-machi Hachioji Tokyo 192-0015 Japan; e Graduate School of Science and Engineering, Department of Chemistry, Materials and Bioengineering, Kansai University 3-3-35 Yamate-cho Suita Osaka 564-8680 Japan; f Department of Parasitology and Tropical Medicine, Kitasato University School of Medicine 1-15-1 Kitazato, Minami-ku Sagamihara Kanagawa 252-0374 Japan; g Drug Innovation Center Lead Exploration Unit, Graduate School of Pharmaceutical Sciences, Osaka University 1-6 Yamadagaoka Suita Osaka 565-0871 Japan

## Abstract

Correction for ‘Anti-schistosomal activity and ADMET properties of 1,2,5-oxadiazinane-containing compound synthesized by visible-light photoredox catalysis’ by Kennosuke Itoh *et al.*, *RSC Med. Chem.*, 2024, https://doi.org/10.1039/d4md00599f.

The authors deeply regret that an essential related study (shown below as ref. 1) was not cited in ref. 46 in the originally published version, and wish to include it alongside the existing references to enhance the comprehensiveness of the paper.

The Royal Society of Chemistry apologises for these errors and any consequent inconvenience to authors and readers.
